# Type 2 diabetes remission after Roux-en-Y gastric bypass (RYGB), sleeve gastrectomy (SG), and one anastomosis gastric bypass (OAGB): results of the longitudinal assessment of bariatric surgery study

**DOI:** 10.1186/s12902-022-01171-8

**Published:** 2022-10-26

**Authors:** Marziyeh Moradi, Ali Kabir, Davood Khalili, Maziar Moradi Lakeh, Masoud Solaymani Dodaran, Abdolreza Pazouki, Mohammad Kermansaravi, Peyman Alibeigi, Hashem Moazenzadeh, Mohammad Reza Abdolhosseini, Foolad Eghbali, Hamid Reza Baradaran

**Affiliations:** 1 Department of Epidemiology, School of Public Health, Iran University of Medical Sciences, Tehran, Iran; 2 Minimally Invasive Surgery Research Center, Iran University of Medical Sciences, Tehran, Iran; 3 Prevention of Metabolic Disorders Research Center, Research Institute for Endocrine Sciences, Shahid Beheshti University of Medical Sciences, Tehran, Iran; 4 Preventive Medicine and Public Health Research Center, Department of Community and Family Medicine, School of Medicine, Psychosocial Health Research Institute, Iran University of Medical Sciences, Tehran, Iran; 5 Department of Surgery, Minimally Invasive Surgery Research Center, Division of Minimally Invasive and Bariatric Surgery, Rasool-e Akram Hospital, Iran University of Medical Sciences, Tehran, Iran; 6 Department of Surgery, Minimally Invasive Surgery Research Center, Division of Minimally Invasive and Bariatric Surgery, Rasool-e Akram and Nikan Hospital, Iran University of Medical Sciences, Tehran, Iran; 7 General Surgeon, Erfan Hospital, Tehran, Iran; 8 Ageing Clinical & Experimental Research Team, Institute of Applied Health Sciences, University of Aberdeen, AB25 2ZD Aberdeen, Scotland, UK; 9 Endocrine Research Center, Institute of Endocrinology and Metabolism, Iran University of Medical Sciences, Tehran, Iran

**Keywords:** Bariatric surgery, Remission, Type 2 diabetes mellitus, Obesity

## Abstract

**Background:**

Several studies on various bariatric surgeries involving patients with type 2 diabetes mellitus (T2DM) showed an overall rate of remission of hyperglycemia. However, there is little known about predictive factors on remission after different types of surgeries. The aim of this study was to identify the T2DM remission rate and to determine the effects of preoperative factors characteristics of remission of type 2 diabetes in Iran.

**Methods:**

We conducted a retrospective analysis of 1351 patients with T2DM operated by three different types of surgeries (Roux-en-Y gastric bypass (RYGB), sleeve gastrectomy (SG), and One Anastomosis Gastric Bypass (OAGB)). Diabetes remission was defined according to the American Diabetes Association (ADA) criteria. Binary logistic regression analyses were employed.

**Results:**

A total of 1351 patients, 675 patients (50.0%) undergoing OAGB, 475 (35.2%) RYGB, and 201 (14.9%) SG. 80.6%, 84.2% of OAGB, 81.7%, 82.6% of RYGB, and 77.1%, 81.5% of SG participants were in T2DM remission after 1 and 3 years, respectively. 1- and 3-year remission were associated with preoperative age, duration of T2DM, FBS and HbA1c, BMI, insulin therapy, and a family history of obesity (p < 0.05).

**Conclusion:**

The remission of T2DM after RYGB, SG, and OAGB surgery is dependent on various preoperative factors. Patients with younger age, shorter duration of T2DM, lower preoperative HbA1c and FBS, higher BMI, who were not on insulin therapy, and not having a family history of obesity were the best candidates to achieve a prolonged diabetes remission.

## Background

Obesity is one of the most common public health concerns with more than 650 million adults being obese. The worldwide prevalence of type 2 diabetes mellitus (T2DM) nearly tripled between 1975 and 2016 and also rose alongside obesity with more than 422 million people suffering from T2DM and 1.5 million deaths directly attributed to diabetes each year. This has been commonly referred to as ‘diabesity’. Hence the prevention and treatment of diabesity is an important public health priority [1]. Patients with T2DM in Iran suffer from relatively poor health-related quality of life and more than 8.7% of total health expenditure in the Iran healthcare system consume by T2DM [2, 3].

American Diabetes Association and the recent International Federation for the Surgery of Obesity and Metabolic Disorders- Asia Pacific Chapter (IFSO-APC) statement have approbated bariatric surgery as the most effective treatment for T2DM or metabolic syndrome for patients who are inadequately controlled by lifestyle alternations and medical treatment for acceptable Asian candidates with BMI ≥ 30 [4, 5]. Several high-quality evidence from randomized controlled trials has demonstrated superior benefits of bariatric surgery to conventional medical therapy in diabetes remission [6–8].

Bariatric surgery can also treat most obesity-related co-morbidities, such as cardiovascular disease, obstructive sleep apnea, hypertension, dyslipidemia, and asthma [9].

Although there is a substantial improvement in glycemic control of diabetic patients who underwent bariatric surgery, not all patients with T2DM achieve remission after bariatric surgery [10]. Many preoperative patient factors, such as age, the use of insulin, body mass index (BMI), glycosylated hemoglobin (HbA1C), diabetes duration, fasting c-peptide level, and medication usage were associated with T2DM remission [11–17].

It is important to note that not all people who undergo bariatric surgery derive equal benefits from surgery. Therefore, identifying preoperative predictors of T2DM remission is needed to determine who is likely to meet T2DM remission criteria and obtain the best results after surgery [18].

The aim of this study was to determine the status of T2DM following three different types of procedures (Roux-en-Y gastric bypass (RYGB), sleeve gastrectomy (SG), and One Anastomosis Gastric Bypass (OAGB)) and also, the preoperative patient characteristics that predict the remission of T2DM after bariatric surgery in patients with BMI ≥ 30 kg/m^2^.

## Methods

We performed a retrospective analysis of a bariatric surgery cohort of patients with T2DM with BMI ≥ 30 kg/m^2^ operated by three different types of surgery (RYGB, SG, and OAGB) from August 2009 to February 2021. All patients were operated on by six surgeons. The diagnosis and classification of T2DM was determined using the criteria established by the American Diabetes Association (ADA) [19].

Relevant demographic and metabolic data were retrieved from a prospectively national obesity surgery database [20]. The study was approved by the institutional review board and ethics committee. Each patient signed an informed consent form at the first preoperative visit for using their data in such studies.

The primary outcome of this study was the rate of diabetes remission based on the following definitions. Diabetes remission was assessed at 1year and 3 years postoperatively. The secondary outcome was to identify predictors of T2DM remission after bariatric surgery.

### Definition of T2DM remission

This study used the definition and interpretation of remission in T2DM after bariatric surgery according to the American Society for Metabolic and Bariatric Surgery (ASMBS) criteria [21]. Complete remission was defined as HbA1c < 6% and FBG < 100 mg/dl in the absence of antidiabetic medications. Partial remission was defined as HbA1c 6-6.4% and FBG 100–125 mg/dl in the absence of antidiabetic medications. Improvement was defined as a statistically significant reduction in HbA1c (by > 1%) and FBG (by > 25 mg/dl) not meeting the criteria for remission or if there was a significant reduction in antidiabetic medications or dose (by discontinuing insulin or one oral agent or 1/2 reduction in dose). Patients were considered unchanged if there was no remission or improvement criteria as described earlier. Recurrence was defined as FBG or HbA1c in the diabetic range (≥ 126 mg/dl and ≥ 6.5%, respectively) or the need for antidiabetic medications after any period of complete or partial remission.

## Statistical analyses

Continuous variables were presented as mean ± SD (standard deviation) or median (interquartile range, IQR). Categorical variables were expressed as frequencies and percentages. Normality was assessed by Shapiro-Wilk’s test. Paired t-test and one-way analysis of variance (ANOVA) with post hoc Bonferroni test was used for parametric data, and Kruskal-Wallis test for nonparametric data. Pearson’s chi-squared test was used for categorical variables.

We defined a composite binary outcome of diabetes remission (complete and partial remission were considered diabetes remission and others were considered non-remission). The association between T2DM remission at 1-year and 3 years after bariatric surgery and various preoperative characteristics were analyzed using binary logistic regression analysis (odds ratio with 95% confidence interval). The enter method was used for variable selection. A P-value < 0.05 was considered statistically significant. Missing data at independent variables were estimated using the Multiple Imputation with Chained Equations (MICE) method. This is commonly used to impute missing covariate (predictor) data [22, 23]. All statistical analyses were performed using Stata software version 12.0.

## Results

A total of 14,391 members of the national obesity surgery database underwent bariatric surgery during the study period. Of them, 1351 patients (9.4%) were with T2DM. The surgeries performed were 675 (50.0%) OAGB, 475 (35.2%) RYGB, and 201 (14.9%) SG. Table 1 presents the demographic and preoperative characteristics of the patients according to type of surgery. 1351 and 853 patients completed 1- and 3-year follow-up, respectively. During the first-year follow-up, 5 patients died, and during the third-year follow-up, 4 patients died. The median preoperative alanine aminotransferase (ALT) in patients who underwent RYGB was higher than OAGB and SG (p = 0.028). There was a significant difference in the prevalence of cardiovascular disease (p < 0.001), dyslipidemia (p = 0.040) and fatty liver (p = 0.008) between patients in three type of bariatric surgery.

**Table 1:** Preoperative characteristics of patients according to type of bariatric surgery

Characteristic	Preoperative			p-value
	**RYGB** **(n = 475)**	**OAGB** **(n = 675)**	**SG** **(n = 201)**	
Gender				
Female, n (%) Male, n (%)	372 (78.3) 103 (21.7)	522 (77.3) 153 (22.7)	161 (80.1) 40 (19.9)	0.700^a^
Age on day of surgery (years)	47.2 ± 9.6	47.3 ± 10.2	47.5 ± 10.9	0.905^b^
Diabetes duration (years)	5 (2–9)	5 (2–9)	5 (3–10)	0.332^c^
BMI (kg/m^2^)	43.7 ± 6.7	44.6 ± 7.0	43.8 ± 6.9	0.071^b^
Body Weight (Kg)	115.5 ± 21.7	117.3 ± 22.7	114.9 ± 21.4	0.227^b^
Insulin use, n (%)	134 (28.2)	186 (27.6)	65 (32.3)	0.413^a^
Number of oral antidiabetic medications before surgery, n (%)				
0 1 2 ≤3	88 (18.5) 283 (59.6) 86 (18.1) 18 (3.8)	143 (21.2) 398 (59.0) 112 (16.6) 22 (3.2)	40 (19.9) 112 (55.7) 43 (21.4) 6 (3.0)	0.712^a^
Fasting blood sugar (mg/dl)	168.4 ± 66.1	166.4 ± 59.9	173.1 ± 67.9	0.422^b^
HbA1c (%)	7.7 ± 1.8	7.8 ± 1.8	7.8 ± 1.8	0.939 ^b^
Total cholesterol (mg/dl)	189.0 ± 53.7	187.1 ± 43.1	182.2 ± 47.2	0.237 ^b^
Triglyceride (mg/dl)	202.9 ± 119.2	192.6 ± 105.1	192.3 ± 105.9	0.259 ^b^
HDL-cholesterol (mg/dl)	44.7 ± 11.3	44.7 ± 10.2	43.7 ± 9.6	0.422 ^b^
LDL- cholesterol (mg/dl)	108.0 ± 41.2	106.4 ± 36.0	103.0 ± 37.9	0.303 ^b^
AST (IU/L)	23 (16–32)	21 (16–30)	23 (17–34)	0.151 ^c^
ALT (IU/L)	29 (19–46)	26 (18–38)	27 (17–43)	**0.028** ^c^
Systolic blood pressure, mm Hg	128.9 ± 17.0	128.3 ± 15.5	128.5 ± 16.8	0.782 ^b^
Diastolic blood pressure, mm Hg	82.3 ± 10.9	82.3 ± 9.8	81.7 ± 9.9	0.746 ^b^
Hypertension, n (%)	207 (43.6)	278 (41.2)	84 (41.8)	0.717 ^a^
Cardiovascular Disease, n (%)	98 (20.6)	204 (30.2)	73 (36.3)	**< 0.001** ^a^
Dyslipidemia, n (%)	212 (44.6)	333 (49.3)	80 (39.8)	**0.040** ^a^
Hypothyroidism, n (%)	128 (26.9)	202 (29.9)	58 (28.9)	0.546 ^a^
Sleep Apnea, n (%)	166 (34.9)	200 (29.6)	69 (34.3)	0.129 ^a^
Asthma, n (%)	44 (9.3)	60 (8.9)	23 (11.4)	0.548 ^a^
Fatty Liver, n (%)	388 (81.7)	593 (87.8)	165 (82.1)	**0.008** ^a^
Family History of, n (%)				
Obesity Hypertension T2DM Dyslipidemia Fatty Liver	152 (32.0) 25 (5.3) 341 (71.8) 6 (1.3) 42 (8.8)	243 (36.0) 53 (7.8) 481 (71.3) 9 (1.3) 53 (7.8)	69 (34.3) 18 (8.9) 128 (63.7) 3 (1.5) 16 (7.9)	0.372 ^a^ 0.132 ^a^ 0.081 ^a^ 0.972 ^a^ 0.826 ^a^

Data are shown as mean ± standard deviation for variables with normal distribution and as median (IQR) for variables with skewed distribution and number (%) for categorical variables

^a^The result is based on the chi-square test

^b^The result is based on the ANOVA test

^c^The result is based on the Kruskal-Wallis test

### Clinical outcomes

A significant difference was noticed between the preoperative and postoperative values of mean weight, BMI, FBS, HbA1c, total cholesterol, triglyceride, HDL, LDL at 1-year and 3 years after surgery. Reduction in the number of patients who were on insulin therapy before surgery from 385(28.5%) to 72(5.4%) at 1 year and 40(4.7%) at 3 years after surgery, and cessation of oral antidiabetic medications in most patients 1 year and 3 years after surgery was found to be statistically significant (Table 2).

**Table 2:** Postoperative changes after 1 and 3 years

Characteristic	Preoperative (n = 1351)	Postoperative 1 year (n = 1351)	P-value^a^	Postoperative 3 years (n = 853)	P-value^b^
BMI (kg/m^2^)	44.1 ± 6.9	30.2 ± 5.3	< 0.001	30.9 ± 4.7	< 0.001
Body Weight (Kg)	116.3 ± 22.2	79.3 ± 15.8	< 0.001	81.2 ± 15.8	< 0.001
Fasting blood sugar (mg/dl)	168.1 ± 63.4	93.7 ± 20.7	< 0.001	98.4 ± 22.4	< 0.001
HbA1c (%)	7.8 ± 1.8	5.7 ± 1.1	< 0.001	5.8 ± 1.2	< 0.001
Total cholesterol (mg/dl)	187.1 ± 47.7	171.9 ± 47.3	0.002	177.1 ± 40.2	0.002
Triglyceride (mg/dl)	196.3 ± 111.2	117.6 ± 62.9	< 0.001	107.6 ± 43.0	< 0.001
HDL-cholesterol (mg/dl)	44.6 ± 10.6	53.7 ± 20.6	< 0.001	52.3 ± 11.0	< 0.001
LDL- cholesterol (mg/dl)	106.5 ± 38.2	96.0 ± 38.1	0.041	103.6 ± 35.8	0.041
Insulin use, n (%)	385 (28.5)	72 (5.4)	< 0.001	40 (4.7)	< 0.001
Number of oral antidiabetic medications before surgery, n (%)					
0 1 2 ≤3	271 (20.1) 793 (58.7) 241 (17.8) 46 (3.4)	1161 (85.9) 161 (11.9) 25 (1.9) 4 (0.3)	< 0.001	749 (87.8) 79 (9.3) 22 (2.6) 3 (0.3)	< 0.001

Data are shown as mean ± standard deviation for continuous variables and number (%) for categorical variables

^a^ Preoperative versus 1 year

^b^ Preoperative versus 3 years

### Weight reduction

Mean BMI ± SD at baseline, 1 year, and 3 years postoperatively were 44.6 ± 7.0, 30.0 ± 5.0 (32.7% weight loss [WL] from baseline weight), and 30.6 ± 4.6 (31.4% WL from baseline weight) for OAGB; 43.7 ± 6.7, 30.2 ± 5.1 (30.9% WL), and 31.3 ± 4.9 (28.4% WL) for RYGB; and 43.8 ± 6.9, 31.9 ± 6.8 (27.2% WL), and 30.8 ± 4.7 (29.7% WL) for SG (Table 3). During the first year of follow-up, mean BMI decreased by 14.6, 13.5, and 11.9 units, for OAGB, RYGB, and SG, respectively. At 3 years of follow-up, reduction in the mean BMI, from baseline, were approximately same among the surgeries: 14.0, 12.4, and 13.0, respectively (Table 3).

**Table 3:** Trends in BMI over time, according to type of surgery

	Preoperative^*^	Postoperative 1 year^*^	Postoperative 3 year^*^
OAGB	44.6 ± 7.0	30.0 ± 5.0	30.6 ± 4.6
RYGB	43.7 ± 6.7	30.2 ± 5.1	31.3 ± 4.9
SG	43.8 ± 6.9	31.9 ± 6.8	30.8 ± 4.7

At 1 year and 3 years follow-up, for all surgeries, postoperative BMI versus preoperative are different at the level of p < 0.05

*mean ± SD

### Glucose control

**Fig. 1:**
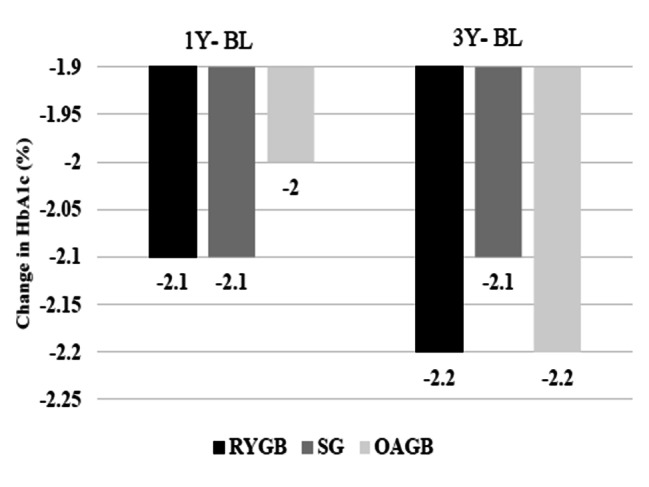
HbA1c change over time, according to the surgeries. Changes in HbA1c between baseline (BL) and 1 year (1Y) postoperatively, BL and 3Y for RYGB, OAGB, and SG surgeries

For the three surgeries, a mean reduction of HbA1c of 2.0% or more was maintained at all two-time points. Mean HbA1c decreased by 2.1% in the first year following RYGB and SG, and by 2% following OAGB. The mean HbA1c decreased slightly more following three types of surgeries during the third year postoperatively (Fig. 1).

### Diabetes remission

Complete remission of T2DM was achieved in a total of 1010 (74.8%) patients at 1-year, and 3 years of prolonged remission was achieved in 677 (79.4%) patients as shown in Table 4. At 1-year follow-up, complete remission rate was approximately the same for patients who underwent RYGB and OAGB, and was higher than compared to SG, but not statistically significant. During 3 years, rates of complete remission increased for patients who underwent RYGB, OAGB and SG.

**Table 4:** T2DM remission 1 and 3 years after RYGB, OAGB, and SG surgery

Diabetic status, n (%)	Postoperative 1-year (n = 1351)					Postoperative 3 years (n = 853)				
	**ALL**	**RYGB**	**OAGB**	**SG**	**P-value**	**ALL**	**RYGB**	**OAGB**	**SG**	**P-value**
Complete remission	1010 (74.8)	359 (75.6)	509 (75.4)	142 (70.6)	0.590	677 (79.4)	247 (79.4)	336 (80.4)	94 (75.8)	0.695
Partial remission	77 (5.7)	29 (6.1)	35 (5.2)	13 (6.5)		33 (3.9)	10 (3.2)	16 (3.8)	7 (5.7)	
Improvement	196 (14.5)	63 (13.3)	97 (14.4)	36 (17.9)		72 (8.4)	29 (9.3)	30 (7.2)	13 (10.5)	
Unchanged	65 (4.8)	24 (5.1)	31 (4.6)	10 (5.0)		54 (6.3)	21 (6.8)	27 (6.5)	6 (4.8)	
Recurrence	3 (0.2)	0	3 (0.4)	0		17 (2.0)	4 (1.3)	9 (2.1)	4 (3.2)	

p-values represent association among all three groups

### Predictors of T2DM remission

Univariable and multivariable associations with the probability of remission at 1-year and 3 years after surgery are shown in Tables 5 and 6. In univariable analysis, age, preoperative BMI, duration of T2DM, no insulin use, FBS, HbA1c, and no family history of obesity were the best preoperative predictors of diabetes remission 1-year and 3 years after surgery.

However, when all these variables were fitted into a multivariable logistic regression model, younger age, higher perioperative BMI, shorter duration of T2DM, no insulin therapy, lower preoperative FBS and HbA1c, and not having a family history of obesity were significantly associated with increased the probability of diabetes remission at 1-year and 3 years after surgery (p < 0.05). At 1 and 3 years after surgery, the type of surgery was not a predictive factor for postoperative diabetes remission after adjustment for potential confounders.

**Table 5:** Logistic regression analysis of preoperative factors in patients with and without diabetes remission 1-year after surgery

Characteristic	Unadjusted OR (95% CI)	p-value	Adjusted OR (95% CI) ^*^	p-value
Female (male = ref)	0.79 (0.57–1.12)	0.194	0.68 (0.45–1.02)	0.064
Age on day of surgery (for each year)	0.95 (0.94–0.96)	**< 0.001**	0.97 (0.95–0.98)	**< 0.001**
BMI (kg/m2) (for each 1 unit)	1.05 (1.03–1.08)	**< 0.001**	1.04 (1.01–1.07)	**0.003**
Diabetes duration (for each year)	0.86 (0.84–0.88)	**< 0.001**	0.92 (0.89–0.94)	**< 0.001**
Insulin use	0.16 (0.12–0.22)	**< 0.001**	0.28 (0.19–0.42)	**< 0.001**
Number of oral antidiabetic medications before surgery				
0 1 2 ≥3	1 1.11 (0.59–2.09) 1.04 (0.47–2.29) 0.45 (0.15–1.31)	0.739 0.923 0.141	1 1.06 (0.58–1.94) 0.96 (0.45–2.07) 0.58 (0.15–2.17)	0.857 0.924 0.418
Type of surgeries				
RYGB OAGB SG	1 0.93 (0.68–1.26) 0.76 (0.51–1.13)	0.642 0.173	1 0.84 (0.58–1.22) 0.79 (0.48–1.30)	0.354 0.357
Fasting blood sugar (mg/dl) (for each 1 mg/dl)	0.99 (0.98–0.99)	**< 0.001**	0.995 (0.991–0.998)	**0.001**
HbA1c (for each 1%)	0.64 (0.59–0.69)	**< 0.001**	0.78 (0.69–0.87)	**< 0.001**
Total cholesterol (mg/dl)	1.00 (0.995–1.01)	0.835	1.003 (0.995–1.012)	0.445
Triacylglycerol (mg/dl)	1.00 (0.999–1.001)	0.763	0.998 (0.997-1.00)	0.084
HDL-cholesterol (mg/dl)	1.001 (0.989–1.014)	0.852	1.00 (0.998–1.002)	0.997
LDL- cholesterol (mg/dl)	0.999 (0.995–1.002)	0.410	0.995 (0.986–1.004)	0.282
AST (IU/L)	0.997 (0.99–1.004)	0.391	1.00 (0.986–1.013)	0.943
ALT (IU/L)	0.998 (0.99–1.01)	0.265	0.998 (0.988–1.008)	0.674
Systolic blood pressure, mm Hg	0.999 (0.991–1.007)	0.843	1.006 (0.992–1.019)	0.423
Diastolic blood pressure, mm Hg	1.003 (0.989–1.016)	0.707	0.996 (0.974–1.018)	0.725
Obesity-related disease (no = ref)				
Dyslipidemia Hypertension Cardiovascular Disease Hypothyroidism Sleep Apnea Asthma Fatty Liver	1.06 (0.81–1.39) 1.02 (0.78–1.34) 1.11 (0.82–1.50) 0.83 (0.62–1.11) 0.83 (0.62–1.09) 1.42 (0.86–2.36) 0.96 (0.67–1.40)	0.667 0.869 0.512 0.215 0.187 0.173 0.839	1.13 (0.79–1.60) 1.04 (0.73–1.47) 1.15 (0.76–1.72) 0.69 (0.48–1.02) 0.84 (0.58–1.21) 1.76 (0.94–3.32) 1.21 (0.76–1.93)	0.504 0.834 0.508 0.168 0.339 0.079 0.417
Family History of, (no = ref)				
Obesity Hypertension Diabetes Dyslipidemia Fatty Liver	0.42 (0.26–0.68) 1.34 (0.76–2.36) 0.75 (0.55–1.02) 0.90 (0.12–6.86) 1.14 (0.45–2.88)	**< 0.001** 0.317 0.064 0.919 0.790	0.33 (0.19–0.56) 1.72 (0.82–3.64) 0.95 (0.65–1.39) 0.60 (0.12–3.01) 1.03 (0.44–1.78)	**< 0.001** 0.154 0.813 0.537 0.731

* Multivariable logistic regression including all factors listed in the table

**Table 6:** Logistic regression analysis of preoperative factors in patients with and without diabetes remission 3 years after surgery

Characteristic	Unadjusted OR (95% CI)	p-value	Adjusted OR (95% CI) ^*^	p-value
Female (male = ref)	0.91 (0.58–1.40)	0.657	0.71 (0.42–1.21)	0.206
Age on day of surgery (years)	0.96 (0.94–0.98)	**< 0.001**	0.96 (0.94–0.99)	**0.003**
BMI (kg/m^2^)	1.07 (1.04–1.11)	**< 0.001**	1.07 (1.03–1.11)	**< 0.001**
Diabetes duration (years)	0.88 (0.85–0.91)	**< 0.001**	0.896 (0.87–0.93)	**< 0.001**
Insulin use	0.17 (0.11–0.24)	**< 0.001**	4.679 (2.82–7.76)	**< 0.001**
Number of oral antidiabetic medications before surgery				
0 1 2 ≥3	1 0.94 (0.58–1.49) 0.61 (0.35–1.06) 0.49 (0.16–1.27)	0.782 0.081 0.134	1 0.906 (0.49–1.65) 0.67 (0.33–1.37) 0.49 (0.13–1.84)	0.747 0.272 0.295
Type of surgeries				
RYGB OAGB SG	1 1.12 (0.76–1.66) 0.92 (0.54–1.58)	0.571 0.770	1 1.01 (0.62–1.64) 0.96 (0.49–1.88)	0.974 0.910
Fasting blood sugar (mg/dl)	0.989 (0.986–0.991)	**< 0.001**	0.992 (0.988–0.995)	**< 0.001**
HbA1c (%)	0.64 (0.58–0.71)	**< 0.001**	0.82 (0.71–0.96)	**0.010**
Total cholesterol (mg/dl)	0.999 (0.996–1.002)	0.593	1.01 (0.99–1.02)	0.695
Triacylglycerol (mg/dl)	0.998 (0.997-1.00)	0.202	0.999 (0.996–1.001)	0.326
HDL-cholesterol (mg/dl)	1.01 (0.991–1.03)	0.328	1.01 (0.98–1.03)	0.617
LDL- cholesterol (mg/dl)	0.998 (0.994–1.003)	0.479	0.997 (0.984–1.01)	0.720
AST (IU/L)	0.997 (0.989–1.004)	0.393	0.992 (0.978–1.005)	0.221
ALT (IU/L)	0.996 (0.99–1.002)	0.192	1.005 (0.988–1.022)	0.582
Systolic blood pressure, mm Hg	1.00 (0.98–1.02)	0.906	1.002 (0.983–1.022)	0.807
Diastolic blood pressure, mm Hg	1.01 (0.95–1.03)	0.158	1.021 (0.990–1.05)	0.187
Obesity-related disease (no = ref)				
Dyslipidemia Hypertension Cardiovascular Disease Hypothyroidism Sleep Apnea Asthma Fatty Liver	0.79 (0.55–1.13) 0.94 (0.65–1.36) 1.33 (0.88–2.01) 0.86 (0.58–1.27) 0.89 (0.61–1.31) 1.27 (0.64–2.55) 0.77 (0.45–1.31)	0.190 0.761 0.175 0.443 0.564 0.496 0.334	0.66 (0.42–1.06) 0.85 (0.53–1.34) 1.39 (0.81–2.43) 0.70 (0.42–1.16) 1.001 (0.61–1.65) 1.49 (0.59–3.75) 1.38 (0.71–2.67)	0.085 0.483 0.232 0.168 0.996 0.401 0.342
Family History of, (no = ref)				
Obesity Hypertension Diabetes Dyslipidemia Fatty Liver	0.42 (0.26–0.68) 1.06 (0.58–1.93) 0.96 (0.67–1.04) 3.06 (0.40-23.39) 1.10 (0.64–1.92)	**< 0.001** 0.859 0.829 0.280 0.727	0.46 (0.28–0.76) 1.43 (0.63–3.27) 1.45 (0.89–2.34) 1.16 (0.14–9.82) 1.38 (0.66–2.92)	**0.003** 0.396 0.130 0.895 0.393

* Multivariable logistic regression including all factors listed in the table

## Discussion

The field of metabolic and bariatric surgery has advanced rapidly over the last decade and this is due in large part to the effect of these operations on T2DM. To our knowledge, this study represents the largest cohort in our country focusing on T2DM remission after three types of bariatric surgery (RYGB, SG, OAGB). We showed that bariatric surgery is a surgical procedure to reach the remission of T2DM. In our cohort, 74.8% and 79.4% of patients experienced complete T2DM remission at 1 year and 3 years after bariatric surgery, respectively. Consistently, a retrospective analysis of 114 individuals who underwent RYGB revealed that 47.4% achieved complete T2D remission during a mean follow-up of 12 months [24]. 1-year after surgery, we observed 74.8% complete remission and 5.7% partial remission rates. Complete remission level was more than those reported by other authors (42.2% [25], 68.91% [26], 74% [10]). On the other hand, the T2DM remission rate 3 years after bariatric surgery reported in this study is higher than rates reported in prospective and retrospective other cohort studies (79.4% at 3 years vs. 62-77%) [27, 28]. Such variations in T2DM remission rates are probably because of the heterogeneity of definitions of T2DM remission employed by different studies. For example, others observed a decrease in complete remission from 92.7 to 43.6% when using a more precise definition [29]. Hence, we used standardized outcome reporting for T2DM remission to enable more precise comparisons throughout the medical literature. In our study, there was no statistically significant difference in the remission of type 2 diabetes 1 year and 3 years after surgery according to three types of bariatric surgeries.

Given the invasive nature of bariatric surgery, it is critical to identify patients who can best benefit from surgery. Because by using the most metabolically effective procedure, some diabetic patients still did not reach diabetes remission after surgery [30]. The present study found that, younger patients, with a shorter duration of diabetes, and lower preoperative FBS and Hba1c, with higher preoperative BMI, that were less likely to, took insulin therapy and were less likely to have had a family history of obesity were more likely to have achieved T2DM remission 1-year and 3 years after bariatric surgery.

In terms of patient characteristics, patients who achieved T2DM remission 1-year and 3 years after surgery were likely to be younger, consistent with others [9, 31, 32], and with research where patients who achieved T2DM remission were approximately 6.48 years younger compared with those who did not [33]. Aging is associated with greater deterioration of pancreatic reserve and disease progression resulting in lower remission rates [16]. As for the duration of diabetes, a shorter T2D duration was associated with remission, consistent with research where patients in remission had a shorter mean T2DM duration compared with those with no remission [32]. Longer T2DM duration reflects disease severity because of reduced beta-cell function and secretory capacity, which significantly reduces the chances of remission [31, 34, 35]. Our study showed that patients with higher BMI had higher remission rates. Bhasker et al.‘s study and Dixon et al.‘s study introduced BMI greater than 35 kg/m2 as a predictive variable for type 2 diabetes improvement after surgery[36, 37]. While the study by Mingrone et al., Panunzi et al., and Lee et al. showed that preoperative BMI was not associated to postoperative type 2 diabetes remission [38–40]. Higher BMI is usually associated with greater weight loss and hence, the higher remission rates. We also showed that lower preoperative HbA1c and FBS levels to be positive predictors of diabetic remission after bariatric surgeries. Like in our study, Jindal et al. also revealed lower HbA1c and FBS before surgery to be associated with higher remission rate [41]. In addition, our results showed that the remission rate was greater for patients who were not taking insulin preoperatively. This supports research where patients not requiring insulin exhibited a higher remission rate than those on insulin [32, 42]. Insulin therapy reflects significantly reduced beta-cell function that may not fully respond to the weight loss and increase in incretin after bariatric surgery [31, 32].

Of great interest, we showed that a family history of obesity can play an important role in predicting T2DM remission. To our knowledge, no study attributed this role to the family history of obesity. However, because family history reflects genetic susceptibility in addition to another factor, and because most Iranian families are very large extended families, it may be a useful predictor for T2DM remission.

Although 50% of patients underwent OAGB, 35.2% RYGB, and 14% SG, there was no statistically significant difference between the three types of surgery in terms of remission of T2DM after adjusted for potential confounders. Of course, it should be kept in mind that the patients in the three type of surgeries had significant differences in ALT, the prevalence of cardiovascular diseases, dyslipidemia, and fatty liver.

In our study, 0.22% and 1.99% of patients who achieved complete or partial remission experienced a subsequent relapse of T2DM at 1 year and 3 years after surgery, which is lower than the range of relapse prevalence (17–40%) reported in the few prior studies that evaluated long terms outcomes of T2DM after surgery [43–45].

This study has some limitations. First, though patient data were gathered prospectively, analysis is retrospective with some missing data, especially in laboratory values. We excluded some patients with missing follow-up outcomes from the analysis that would affect outcome assessment and remission rates undoubtedly. Another limitation is that patients were not operated on by a surgeon.

Despite these, the study has strengths. This is the first report of T2DM remission in patients with T2DM with obesity in our country. Also, we evaluated a range of demographic, anthropometric, biochemical, and clinical factors associated with remission among a larger sample size than most other studies [8, 10, 13], and included three types of bariatric surgery patients. Understanding the modifiable factors that influence T2DM remission after bariatric surgery could lead to better strategies to enhance durable remission. Employing the American Society for Metabolic and Bariatric Surgery (ASMBS) definitions, we categorized 4 remission levels (complete remission, partial remission, improvement, unchanged status), unlike others [32, 46, 47]. The use of logistic regression allowed the evaluation of factors that independently predicted T2DM remission.

In summary, the present study demonstrated weight loss and improvement in glucose control and diabetes remission following three types of bariatric surgery. The higher rates of diabetes remission are seen in younger patients with higher preoperative BMI, lower HbA1c and FBS, with shorter duration of T2DM who were not insulin therapy, and not having a family history of obesity at 1-year and 3 years after bariatric surgery.

## Data Availability

The datasets used and analyzed during the current study are available from the corresponding author on reasonable request.

## Abbreviations

T2DM: Type 2 Diabetes Mellitus
RYGB: Roux-en-Y gastric bypass
SG: sleeve gastrectomy
OAGB: One Anastomosis Gastric Bypass
IFSO-APC: International Federation for the Surgery of Obesity and Metabolic Disorders- Asia Pacific Chapter
WL: Weight Loss
BMI: Body mass index
MICE: Multiple Imputation with Chained Equations
ASMBS: American Society for Metabolic and Bariatric Surgery
OR: Odds Ratio
CI: Confidence Interval
